# Impact of family functioning on mental health problems of college students in China during COVID-19 pandemic and moderating role of coping style: a longitudinal study

**DOI:** 10.1186/s12888-023-04717-9

**Published:** 2023-04-12

**Authors:** Lili Liu, Jianbin Chen, Shunwei Liang, Wenwen Yang, Xiaodan Peng, Chengcheng Cai, Andi Huang, Xiayong Wang, Jingbo Zhao

**Affiliations:** 1 Department of Psychology, School of Public Health, Southern Medical University, Guangzhou, China; 2 Mental Health Education and Counseling Center, Guangzhou Academy of Fine Arts, Guangzhou, China; 3 Psychological Counseling Center, Department of Student Affairs, Yunnan University of Chinese Medicine, Yunnan, China; 4 Mental Health Center, School of Public Health, Southern Medical University, Guangzhou, China

**Keywords:** Young adults, COVID-19, Family functioning, Coping styles, Depressive symptoms, Anxiety, Longitudinal study

## Abstract

**Background:**

During the COVID-19 pandemic, college students were required to stay at home and maintain social distance for the entire spring semester of 2020. There is little research on how family functioning influenced mental health problems and how coping styles moderated the relationship between family functioning and mental health problems among college students during their stay-at-home period.

**Methods:**

A total of 13,462 college students (age = 16–29 years) completed four online surveys between February and October 2020, namely the outbreak phase, remission phase, online study phase, and school reopening phase in Guangdong Province, China. Family functioning was assessed by the Family APGAR; coping styles were assessed by the Simplified Coping Style Questionnaire (SCSQ), depression symptoms and anxiety symptoms were evaluated by the Patient Health Questionnaire (PHQ-9) and the Generalized Anxiety Disorder Scale (GAD-7) respectively. Generalized estimating equations were used to assess associations between variables, the logit link function was used to estimate the odds ratio of different subgroups, the Newton–Raphson method was used to estimate parameters, and the Wald test was used to test the main effect and the interaction effect.

**Results:**

The incidence rates of depression increased during the stay-at-home period from 33.87%, 95% CI (29.88%, 38.10%) to 40.08% 95% CI (35.76%, 44.55%) after schools reopened, χ^2^ = 193.68, *p* < 0.001. The incidence rates of anxiety increased from 17.45%, 95% CI (14.59%, 20.73%) to 26.53%, 95% CI (16.94%, 23.67%) over the entire period, χ^2^ = 195.74, *p* < 0.001. The percentages of students with highly functional, moderately dysfunctional and severely dysfunctional family functioning were 48.23%, 43.91 and 7.86% at T1 and 46.20%, 45.28%, and 8.52 at T4, respectively. The percentage of subjects with active coping style was 23.9%, negative coping style was 17.4%, strong response coping was 26.9%, and weak response coping was 31.7%. The incidence rate of depression and anxiety for different family functioning groups varied at different time points, and the interaction effect was significant (χ2 = 52.97, *p* < 0.001 and χ2 = 51.25, *p* < 0.001, respectively). The incidence rate of depression and anxiety for different family functioning groups with different coping styles also varied at different time points, the interaction effect was likewise significant (χ2 = 862.09, *p* < 0.001 and χ2 = 583.29, *p* < 0.001, respectively).

**Conclusions:**

Having a severely dysfunctional family and a negative coping style increase the incidence rates of depression and anxiety. These findings highlight the importance of paying special attention to college students' family functioning and promoting appropriate coping strategies during and after COVID-19.

**Supplementary Information:**

The online version contains supplementary material available at 10.1186/s12888-023-04717-9.

## Introduction

It is now clearly evident that the COVID-19 pandemic has had a psychological and social impact worldwide, and studies have indicated that mental health problems had become a big global issue [1–3]. To prevent the rapid spread of COVID-19, in February 2020 the Chinese government ordered a nationwide closure of all educational institutions and the Ministry of Education suggested “suspending classes without suspending learning.” Students across the country were required to stay at home and study online [4]. University campus life and learning environment play a crucial role in students’ psychological development, peer relationships, and autonomy [5]. The results of a nationwide survey in China show that the COVID-19 pandemic has brought about a high degree of psychological stress to the general population, especially the younger generation [4–8]. During the first month of the COVID-19 breakout, 79.4% and 71.1% of Chinese college students worried about their family members and themselves becoming infected with COVID-19 [9], the fear of the COVID-19 dropped a year after the breakout but the anxiety symptoms rose [10].

Large-scale health disasters intensify not only the psychological stressors but also the basic human need to feel safe, connected, calm, useful, and hopeful [7]. Previous research studying the impact of COVID-19 on an individual’s mental health mainly focused on the epidemiology of depression, anxiety, sleeping problems, suicidal ideation, and PTSD symptoms [11–13], and the mental health of infected people and vulnerable populations such as elderly, children and healthcare workers [14–16]. However, there is a lack of research on the long-term mixed effects of family and coping strategies that affected mental health problems during the COVID-19 pandemic.

Family is one of the most prominent factors that impact mental health, and impaired family environments contribute to the risk for mental health problems (MHP) among family members [17–19]. A nationwide strict lockdown during the pandemic meant long-term home isolation. Research shows a substantial psychological effect of social isolation [20], and a lack of sufficient personal space in the family may contribute to frequent parent–child conflicts and a surge in family pressure or even family violence [21]. Family functioning is defined as the extent to which a family performs as a unit to cope with stressors [22], which is commonly measured by the family APGAR from five parameters: adaptation, partnership, growth, affection, and resolve [23, 24]. Previous studies have shown that family functioning is positively correlated with mental health before and during the pandemic [23, 25, 26], and children growing up in an environment with high family functioning have a low level of MHP [26].

Under the impact of both the COVID-19 pandemic and family pressure, an individual’s coping style plays a significant role between pressure and mental health [27, 28]. Coping style refers to the specific efforts, both behavioral and psychological, that people employ to master, tolerate, reduce, or minimize stressful events [29]. According to the integrated model of the coping process proposed by N Ntoumanis, J Edmunds [30], coping strategies are the result of stress appraisals, which can be influenced by social context and psychological needs.

When an individual faces a stressful situation, different appraisals occur depending upon the evaluation of the stressor. RS Lazarus and S Folkman [31] have identified four different types of appraisals as harm–loss, threat, challenge, and benign. Harm–loss appraisal refers to damage that has already been done and is more likely to induce an emotionally focused negative coping style. Threat appraisal refers to potential for harm or loss, and because the damage is yet to come, an individual may have a strong response that tries all kinds of strategies to cope. Challenge appraisal refers to an opportunity for personal growth, which easily leads one to take an active coping style and focus on the problem itself. Benign appraisal refers to a situation when an individual believes there is no threat, takes no further appraisal, and gives a weak response [30]. Recent cross-sectional studies have indicated that maladaptive coping strategies such as negative coping emerged as a risk factor for mental health during the pandemic [28, 32, 33], and active coping strategies, such as spiritual skills, can be a resource to address mental health issues [33, 34].

Although previous studies have used retrospective designs concerning possible risk factors [35], little research has been done to understand the effect of interaction between social context and coping style, on the mental health of college students during the quarantine period. This study presents the results of longitudinal research to comprehensively describe changes in the mental health states of college students with different family functioning and different coping styles from the beginning until the end of the quarantine period. The objective of this study was to assess how the main social context (family) and coping style together influence the mental health problems of college students during and after the stay-at-home period. We hypothesize a moderator model, the family functioning would be negatively associated with MHP (hypothesis 1), and the influence will be weakened after schools reopened (hypothesis 2). Different coping styles would moderate the relationship between family functioning and MHP during different times (hypothesis 3).

## Methods

### Study design and participants

This longitudinal prospective observational study was conducted on a large sample of college students from 22 colleges and universities in the Guangdong Province of China. There are 160 colleges and universities in Guangdong Province of China, including 67 undergraduate universities and 93 three-year colleges, we used the representative sampling method to choose the sample schools, which contain 10 undergraduate universities and 12 three-year colleges. The study was carried out in four survey periods: February 3–10, 2020 (T1); March 24 to April 3, 2020 (T2); June 1–15, 2020 (T3); and September 10 to October 17, 2020 (T4).

At the first survey, students had vacations and all colleges were closed. During the second and third survey, students were still at home but taking online courses. At the fourth survey, all colleges were reopened, students were back at school, so it was conducted at each college, using the same means as for the first three surveys.

A total of 164,101 students (response rate 37.3%, valid questionnaire: 88.3%) completed the initial survey (T1) at the first outbreak phase of the pandemic, 148,343 students (response rate 33.7%, valid questionnaire: 95.4%) completed the second-wave survey (T2) at the COVID-19 remission stage (for epidemiologic assessment of the first two surveys, see Y Li, J Zhao, Z Ma, LS McReynolds, D Lin, Z Chen, T Wang, D Wang, Y Zhang, J Zhang, et al. [12]), 159,187 students (response rate 36.2%, valid questionnaire: 95.7%) completed the third-wave survey (T3) at online-study stage, and 120,190 students (response rate 27.3%, valid questionnaire: 97.5%) completed the fourth-wave survey (T4) at school-reopened stage. Of the total participants, 13,462 students who participated in all four surveys were included in further analysis.

This study was approved by the Biomedical Ethics Committee, Southern Medical University, Guangzhou, China. Electronic informed consents were obtained online. All ethical concerns were maintained strictly.

### Procedures

We prepared one common normative notice for all the 22 colleges, which mentioned the purpose, significance, deadline, and mode of participation in the online survey for all the four time periods. All students in the target universities were regarded as potential participants and were asked to voluntarily participate in the survey through the network platform (http://www.togx.cn/step_50.html). (For more details, see prior study Y Li, J Zhao, Z Ma, LS McReynolds, D Lin, Z Chen, T Wang, D Wang, Y Zhang, J Zhang, et al. [12]). We then matched the four data using the student ID to obtain the longitudinal sample data.

### Measurements

#### Demographic information

The demographic information of the participants included age, gender (male or female), and college year (freshman, sophomore, junior, senior, and graduate).

#### Family functioning

The family functioning of the participants was assessed by Family APGAR [24] for T2—T4. Which had been translated into Chinese and have a good validation [36]. Sample items include “I am satisfied that I can turn to my family for help when something is troubling me,” and “I am satisfied with the way my family talks over things with me and shares problems with me.” The scale is rated on a 3-point Likert scale and consists of five items, with each item scored from 0 (*never or rarely*) to 2 (*most or all of the time*). The total score ranges from 0 to 10, and a score of 7—10 indicates a highly functional (HF) family, a score of 4—6 indicates a moderately dysfunctional family (MdF), and a score of 0—3 indicates a severely dysfunctional (SdF) family. The Cronbach α was 0.89 at T2, 0.91 at T3, and 0.90 at T4 in this study.

#### Coping style

The coping style of the participants was assessed using the Simplified Coping Style Questionnaire (SCSQ) [37] at T1. The SCSQ was developed by Chinese scholars and has good validity and applicability [28]. The SCSQ is rated on a 4-point Likert scale and consists of 20 items, with each item scored from 0 (*never*) to 3 (*very often*). It consists of two dimensions: active coping and negative coping. Sample items of active coping include “trying to see things in as good a way as possible” and “finding different ways to solve problems.” The negative coping dimension includes items such as “relieving troubles by smoking and drinking” and “fantasizing that a miracle may happen to change the *status quo*.” The SCSQ score reflects the coping style preferences of participants, with a higher score indicating a higher possibility that the participant would adopt the relevant coping style. To identify the various coping styles theorized by N Ntoumanis, J Edmunds and JL Duda [30], in this study we used the standard score of active/ negative coping to distinguish the participants into four groups using method adapted from Fu et.al [28]. The standard score was achieved by *Z*-transformation of the mean and standard deviation of the active and negative coping styles of the entire sample of T1. If the *Z* score of active coping > 0 and the *Z* score of negative coping ≤ 0, it indicated that the individual generally adopted an active coping style. If the *Z* score of active coping ≤ 0 and the *Z* score of negative coping > 0, it indicated that the individual generally adopted a negative coping style. If the *Z* scores of both active and negative copings > 0, it indicated that the individual generally adopted a strong response coping style. If the *Z* scores of both active and negative copings ≤ 0, it indicated that the individual generally adopted a weak response coping style. The SCSQ is commonly used in China, and the Cronbach α coefficients for the two dimensions were 0.90 and 0.77, respectively, in this study.

#### Mental health issues

Mental health issues reported by participants included depression and anxiety. Depression symptoms were assessed using the Patient Health Questionnaire (PHQ-9) [38] on a 4-point scale ranging from 0 to 3. As validated in a Chinese population, a summed score of 7 indicates probable clinical depression [39]. The Cronbach α was 0.87 at T1, 0.90 at T2, 0.91 at T3, and 0.92 at T4.

Anxiety symptoms were measured using the Chinese version of the Generalized Anxiety Disorder Scale (GAD-7) [40], which consists of 7 items rated on a 4-point scale from 0 to 3. As validated in a Chinese population, a cutoff total score of 7 indicates clinical levels of anxiety [41]. The Cronbach α was 0.91 at T1, 0.92 at T2, 0.94 at T3, and 0.94 at T4.

### Covariates

To control for confounding in the association between family functioning and MHP, the following variables were adjusted: age group (< 18, 18—19, 20—21, 22—23, ≥ 24 years), self-reported gender (male/female), self-reported mental health status before the outbreak, including self-reported prior mental health problems: “Have you ever been diagnosed with a mental illness. (yes/no)”, and self-reported psychological counseling experience: “Have you received psychological counseling services from a professional (counselor, psychiatrist, etc.) in the past (yes/no)”. School type was initially coded as medical university, normal university, multi-faculty university, three-year normal college, and three-year vocational college, but was changed to binary (university and three-year college) in order to reduce the parameters in the statistical models to meet convergence criteria.

### Statistical analysis

A generalized estimating equation (GEE) was used to quantify the change in anxiety and depression through 6 months from the beginning of the COVID-19 pandemic until schools reopened. GEE is commonly used for longitudinal studies with repeated measures [42–44]. The logit link function was used to estimate the odds ratio of different subgroups, the Newton–Raphson method was used to estimate parameters, and the Wald test was used to test for main and interaction effects. The dependent variables were depression (PHQ-9 score ≥ 7) and anxiety (GAD-7 score ≥ 7) which were transformed into dichotomous variables by cutoffs and measured at T1 through T4. Independent variables included time (four levels), family functioning (highly functional family, moderately functional family, and severely dysfunctional family) measured at T2-T4, and coping style (active coping, negative coping, strong response coping, and weak response coping) measured at T1. Covariates included age, gender, health status, and school type. Anxiety and depression were analyzed separately. We used two models to examine the associations. Model 1 was using time and family functioning as independent variables. Model 2 was using time, family functioning, and coping style as independent variables. All covariates were controlled in both models. To present the significant result clearly, in this paper we only describe the differences between T1 and T3 and between T3 and T4 because these two survey pairs show the differences between the 4-month home quarantine and before and after schools reopened.

All analyses were performed using Statistical Package for Social Sciences (SPSS) version 25.0.

## Results

### Descriptive characteristics

A total of the 13,462 participants included in this analysis, 74.8% were female participants aged 16—29 years (*M* = 19.68, SD = 1.38). The percentages of participants with highly functional, moderately dysfunctional and severely dysfunctional family functioning were 48.23%, 43.91% and 7.86% at T1 and 46.20%, 45.28% and 8.52% at T4, respectively. The percentage of subjects with active coping style was 23.9%, negative coping style was 17.4%, strong response coping was 26.9%, and weak response coping was 31.7%. Table 1 presents detailed sample characteristics, including gender, age, college year, university, and college type, ever received counseling for MHP from a professional, and prior MHP.

**Table 1:** Sample characteristics (*N* = 13,462)

Variable	No. of students (%)
**Gender**	
Male	3387 (25.2)
Female	10,075 (74.8)
**Age (years)**	
< 18	918 (6.82)
18–19	7539 (56.00)
20–21	4426 (32.88)
22–23	405 (3.01)
≥ 24	174 (1.29)
**University/college type**	
Medical university	704 (5.23)
Normal university	3421 (25.41)
Multifaculty university	2571 (19.10)
Three-year normal college	2087 (15.50)
Three-year vocational college	4679 (34.76)
**College year**	
Freshman	6924 (51.43)
Sophomore	4483 (33.30)
Junior	1912 (14.20)
Senior	143 (1.07)
**Psychological counseling experience**	
Never	12,957 (96.25)
Yes	505 (3.75)
**Prior mental health problems**	
No	13,381 (99.40)
Yes	81 (0.60)

Table 2 shows sample sizes and percentages of each subgroup categorized by three independent variables: time, family functioning, and coping style. All subgroups had over 100 participants. SdF with active coping style was the smallest group (*n* = 118 at T1-T2, *n* = 172 at T3 and *n* = 147 at T4), and HF with active coping style were the largest group (*n* = 2085 at T1-T2, *n* = 1797 at T3 and *n* = 1949 at T4). Most of the HF and MdF subgroups had around 1000 participants (*n* = 795—2085 participants), whereas SdF subgroups had around 200 participants (*n* = 118—400). The sample sizes were sufficient to proceed to the next statistical step.

**Table 2:** Sample Sizes of Subgroups

	**T1**			**T2**			**T3**			**T4**		
	**N**	**%**^**a**^		**N**	**%**^**a**^		**N**	**%**^**a**^		**N**	**%**^**a**^	
HF	6493	48.23		6493	48.23		5474	40.66		6219	46.20	
Active coping	2085	15.49	(32.11)	2085	15.49	(32.11)	1797	13.35	(32.83)	1949	14.48	(31.34)
Negative coping	795	5.91	(12.24)	795	5.91	(12.24)	629	4.67	(11.49)	782	5.81	(12.57)
Strong response coping	1998	14.84	(30.77)	1998	14.84	(30.77)	1714	12.73	(31.31)	1951	14.49	(31.37)
Weak response coping	1615	12.00	(24.87)	1615	12.00	(24.87)	1334	9.91	(24.37)	1537	11.42	(24.71)
MdF	5911	43.91		5911	43.91		6674	49.58		6096	45.28	
Active coping	1021	7.58	(17.27)	1021	7.58	(17.27)	1255	9.32	(18.80)	1128	8.38	(18.50)
Negative coping	1220	9.06	(20.64)	1220	9.06	(20.64)	1352	10.04	(20.26)	1242	9.23	(20.37)
Strong response coping	1419	10.54	(24.01)	1419	10.54	(24.01)	1625	12.07	(24.35)	1439	10.69	(23.61)
Weak response coping	2251	16.72	(38.08)	2251	16.72	(38.08)	2442	18.14	(36.59)	2287	16.99	(37.52)
SdF	1058	7.86		1058	7.86		1314	9.76		1147	8.52	
Active coping	118	0.88	(11.15)	118	0.88	(11.15)	172	1.28	(13.09)	147	1.09	(12.82)
Negative coping	327	2.43	(30.91)	327	2.43	(30.91)	361	2.68	(27.47)	318	2.36	(27.72)
Strong response coping	211	1.57	(19.94)	211	1.57	(19.94)	289	2.15	(21.99)	238	1.77	(20.75)
Weak response coping	402	2.99	(38.00)	402	2.99	(38.00)	492	3.65	(37.44)	444	3.30	(38.71)

*HF* Highly functional family, *MdF* Moderately functional family, *SdF* Severely dysfunctional family

^a^Values within parentheses indicate within-group percentage

### Longitudinal effect of family functioning on anxiety and depression

Table 3 shows the main effects of time and family functioning on the incidence rates of anxiety and depression. All main effects are significant at *p* < 0.001 level, indicating that the incidence rates of anxiety and depression varied across the four-time points and different family functioning. For the four-time survey periods, the highest incidence rate of depression is 43.53% (95% CI, 39.08—48.08) at T3 (i.e., when the students were at home quarantine), whereas the highest incidence rate of anxiety is 26.53% (95% CI, 22.64—30.82) at T4 (i.e., after students returned to schools). The incidence rates of anxiety and depression were lowest at T1, with 33.87% (95% CI, 29.88—38.10) for depression and 17.45% (95% CI, 14.59—20.73) for anxiety. Among the family functioning groups, the SdF group had the highest incidence rate of both anxiety and depression, with depression at 53.80% (95% CI, 48.98—58.55) and anxiety at 30.20% (95% CI, 25.79—35.00).

**Table 3:** Main Effect of Time and Family Functioning with Marginal Incidence Rates of MHP

Subgroup	Depression % (95% Wald CI)	Anxiety % (95% Wald CI)
**Time**		
T1	33.87 (29.88, 38.10)	17.45 (14.59, 20.73)
T2	39.21 (34.92, 43.67)	20.09 (16.94, 23.67)
T3	43.53 (39.08, 48.08)	22.50 (19.05, 26.37)
T4	40.08 (35.76, 44.55)	26.53 (22.64, 30.82)
Main effect of time Wald χ^2^	193.68***	195.74***
**Family functioning**		
HF	22.46 (19.43, 25.81)	11.20 (9.26, 13.48)
MdF	44.01 (39.62, 48.50)	27.21 (23.29, 31.52)
SdF	53.80 (48.98, 58.55)	30.20 (25.79, 35.00)
Main effect of family functioning Wald χ^2^	1509.21***	985.57***

*MHP* Mental health problems, *HF* Highly functional family, *MdF* Moderately functional family, *SdF* Severely dysfunctional family; ***, *p* < 0.001

Pairwise comparisons of time (see Supplementary Table S1 available online) show that the incidence rates of depression rose during T1-T3 (all significant at the *P* < 0.05 level) and that the incidence rate at T4 is significantly lower than at T3 (*P* < 0.05) but not higher than at T2 (*P* > 0.05). As shown in Table 3, the incidence rates of anxiety rose during T1-T4. Pairwise comparison of family functioning shows that the HF group had the lowest incidence rates and the SdF group had the highest incidence rates for both anxiety and depression (all differences are significant at the *P* < 0.05 level).

Table 4 shows the estimated marginal incidence rates of anxiety and depression of each subgroup divided by time and family functioning. The table also includes the interaction effect and simple effect of anxiety and depression associated with time. The interaction effects are significant at the *p* < 0.001 level for both depression and anxiety, meaning that anxiety and depression changed differently over time for participants with different family functioning.

**Table 4:** Interaction Effects and Simple Effect of Time and Marginal Incidence Rates of MHP

	**Family functioning**			**Time × Family functioning Wald** χ^2^
	**HF**	**MdF**	**SdF**	
**Depression % (95 Wald CI)**				
T1	20.17 (17.27, 23.41)	36.04 (31.88, 40.43)	48.56 (43.20, 53.95)	52.97***
T2	21.19 (18.18, 24.54)	42.74 (38.28, 47.31)	57.21 (51.88, 62.37)	
T3	26.19 (22.68, 30.03)	50.15 (45.56, 54.73)	56.20 (51.01, 61.26)	
T4	22.61 (19.45, 26.11)	47.44 (42.88, 52.04)	53.16 (47.86, 58.39)	
Simple effect of time Wald χ^2^	31.65***	315.37***	53.68***	
**Anxiety % (95 Wald CI)**				
T1	9.86 (8.00, 12.01)	19.43 (16.26, 23.05)	26.45 (21.84, 31.64)	51.25***
T2	10.02 (8.17, 12.24)	24.93 (21.15, 29.12)	30.06 (25.20, 35.43)	
T3	10.99 (8.97, 13.40)	31.13 (26.79, 35.82)	30.48 (25.67, 35.75)	
T4	14.44 (11.92, 17.39)	35.05 (30.41, 39.99)	34.06 (28.88, 39.66)	
Simple effect of time Wald χ^2^	14.43**	220.22***	43.96***	

*MHP* Mental health problems, *HF* Highly functional family, MdF, moderately functional family, *SdF* Severely dysfunctional family. **, *p* < 0.01; ***, *p* < 0.001

The pairwise comparisons of the incidence rates of anxiety and depression for each family functioning group with significant simple effects at different time points (see Supplementary Table S2 available online) show (high and low means significant at *P* < 0.05 level) that for the HF group, the incidence rates of depression do not rise until T3, which is higher than other time, and that T4 is lower than T3 and higher than T1 but not T2. The incidence rates of anxiety rise only at T4, and there is no statistically significant difference between T1 and T3.

In the MdF group, the incidence rates of depression have a significant difference each time, meaning that the growth tendency stops at T4, but is still higher than at T2. The incidence rates of anxiety also have a significant difference between each time, meaning that it continues to grow from T2 to T4.

In the SdF group, the incidence rates of depression are higher at T2 and T3 than at T1 and other differences are not significant. The incidence rates of anxiety are higher at T2—T4 than at T1, and T4 is higher than T2.

**Fig. 1:**
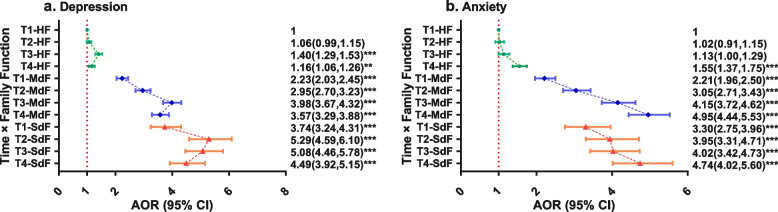
Forest plot showing adjusted multivariance logistic regression analysis: time × family functioning. HF, highly functional family; MdF, moderately dysfunctional family; SdF, severely dysfunctional family; AOR, adjusted odds ratio; **p < 0.01; ***p < 0.001. AOR was adjusted for age, gender, mental health status before the outbreak of the pandemic, and school type

To demonstrate the odds ratio of different subgroups and the change in pattern through time, we carried out an adjusted multivariance logistic regression analysis by EEG, using the T1-HF group as the reference group, covariates mentioned in Sect. 2.4 were controlled in the analyses. Figure 1 shows the forest plot of the adjusted odds ratio (AOR) and 95% CI of each subgroup at the two MHP.

### Longitudinal moderating effect of coping style to family functioning on anxiety and depression

The three-way interaction between time, family functioning, and coping style on depression and anxiety incident rates were calculated, Wald χ^2^ and were 862.09, *p* < 0.001 and 583.29, *p* < 0.001, respectively. The simple effects of time were also calculated (see Table 5 for the simple effect of time by each subgroup). Except HF group with a negative coping style on anxiety, SdF group with negative and weak response coping on both MPH do not have a significant simple effect of time, other subgroups’ MHP are associated with time significantly.

**Table 5:** Interaction Effect and Simple Effect of Time (Wald χ^2^)

Family functioning		*HF*		*MdF*		*SdF*	
**MHP**		**Depression**	**Anxiety**	**Depression**	**Anxiety**	**Depression**	**Anxiety**
**Coping Style**	***Active***	44.92***	17.8***	91.22***	70.88***	39.5***	13.21***
	***Negative***	11.2*	*7.8*	37.42***	64.48***	*4.59*	*5.14*
	***Strong response***	10.04*	15.71***	98.39***	116.83***	16.18***	8.39*
	***Weak response***	18.8***	20.27***	132.24***	99.15***	*5.01*	*1.82*

*HF* Highly functional family, *MdF* Moderately functional family, *SdF* Severely dysfunctional family

*, *p* < 0.05; **, *p* < 0.01; ***, *p* < 0.001

**Fig. 2:**
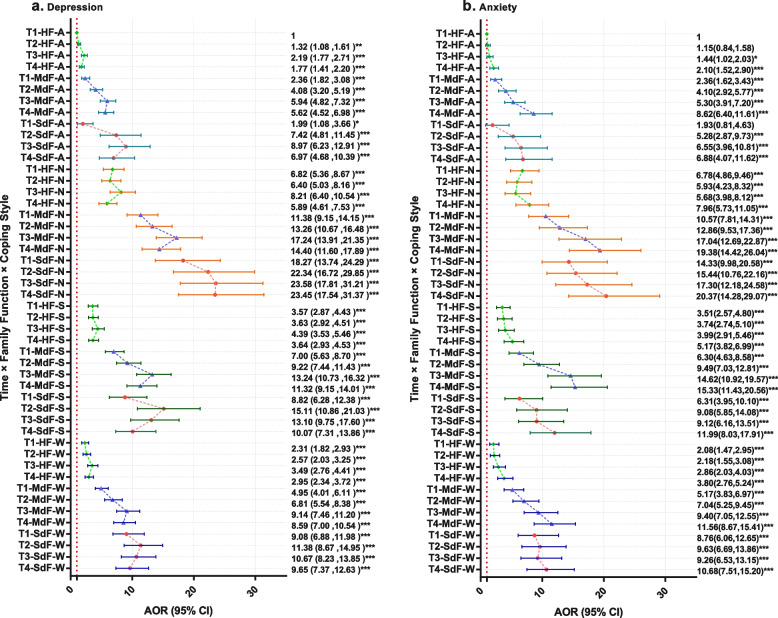
Forest plot showing adjusted multivariance logistic regression analysis: time × family functioning × Coping Style. HF, highly functional family; MdF, moderately dysfunctional family; SdF, severely dysfunctional family; A, active coping style; N, negative coping style; S, strong response coping style; W, weak response coping style; AOR, adjusted odds ratio; **p < 0.01; ***p < 0.001. AOR was adjusted for age, gender, mental health status before the outbreak of the pandemic, and school type

To demonstrate the odds ratio of different subgroups and the change in pattern through time, an adjusted multivariance logistic regression analysis by EEG was carried out using the T1-HF group with active coping style as the reference. Figure 2 shows the forest plot of AOR and 95% CI of each subgroup at the two MHP.

For the HF group, the incidence rates for depression increased between T3 and T1, except for the negative coping subgroup. However, at T3, the AOR of the HF group is 8.21, of the active coping subgroup is 2.19, of the strong response coping subgroup is 4.39, and of the weak response coping subgroup is 3.49; note also that the risk of the negative coping subgroup is high throughout the quarantine period. After school resumed, the incidence rates of MHP were low for all subgroups, except for the weak response subgroup, and the incidence rate of the negative coping subgroup decreased by 7.86% and its AOR decreased to 5.89. The incidence rates for anxiety increased between T3 and T1 for the active and weak response coping subgroups. At T3, the AORs of active and weak response coping subgroups were 1.44 and 2.86, respectively, whereas the AOR of the negative coping subgroup was 5.68. After students returned to school, the incidence rates of all subgroups were higher, and the incidence rate of the negative coping subgroup rose to 5.57%, and the AOR was 7.96.

For the MdF group, the incidence rates of anxiety and depression rose during T1-T3. For the active, negative, strong response, and weak response subgroups, the incidence rates of depression increased to 17.49%, 10.18%, 15.74%, and 14.40%, respectively, and the AORs increased from 2.36 to 5.94, 11.38 to 17.24, 7.00 to 13.24, and 4.95 to 9.14, respectively. For negative and strong response coping style subgroups, the incidence rates of depression fall at T3–T4 by 4.34% and 3.90%, respectively, and the AORs changes from 17.24 to 14.40 for negative coping style and from 13.24 to 11.32 for strong response coping style. For active, negative, strong response, and weak response subgroups, the incidence rates of anxiety rise 8.79%, 10.76%, 16.65%, and 10.13%, respectively, and the AORs rise from 2.35 to 5.3, 10.57 to 17.04, 6.30 to 14.62, and 5.17 to 9.40, respectively. The incidence rates of both depression and anxiety rise at T3 to T4 for the active and weak response coping style group.

For the SdF group, it is noteworthy that the incidence rates of both depression and anxiety did not change after students returned to school. The incidence rates of depression decreased 29.76% and 9.86% from T1 to T3 for active and strong response coping styles, and the AOR rise from 1.99 to 8.97 for active coping style and from 8.8 to 13.10 for strong response coping style. The AOR of depression for the negative coping style increased from 18.27 at T1 to 23.58 at T3, and the AOR for weak response coping style increased from 9.08 at T1 to 10.67 at T3. For the active coping subgroup, the incidence rate of anxiety increased 13.43% from T1 to T3 and the AOR increased from 1.93 to 6.55. The other subgroups did not change significantly from T1 to T3. The AOR of the negative coping subgroup is 14.33 at T1 and 17.3 at T3. For all coping style subgroups, there was no significant change after the students returned to school.

## Discussion

In this longitudinal study, we analyzed the tangling interaction effect of family functioning and coping styles with depression and anxiety, during and after the stay-at-home period from February to October 2020. College students across China were required to take online courses at home for one semester and returned to school to take in-person classes but wear a mask and maintain social distance, it was an opportunity to study the relationship between these variables.

We found that the incidence rates of depression increased from 33.87% (95% CI, 29.88%–38.10%) to 43.53% (95% CI, 39.08%–48.08%) during the stay-at-home period (T1-T3) and decreased to 40.08% (95% CI, 35.76%–44.55%) after school-reopened (T4). The incidence rates of anxiety rose from 17.45% (95% CI, 14.59%–20.73%) to 22.50% (95% CI, 16.94%–23.67%) during the stay-at-home period (T1-T3) and kept rising to 26.53% (95% CI, 22.64%–30.82%) after school-reopened (T4). Our results are in line with the recent studies of M Daly, AR Sutin and E Robinson [8], who state that pronounced and prolonged deterioration in the general mental health of people occurred during the COVID-19 crisis in the UK. The pandemic also has had a negative impact on the mental health of the general population in Spain and the mental health of people still does not seem to be at pre-crisis levels even after the drastic quarantine measures were removed [5]. The prolonged deterioration in mental health after the break out of COVID-19 also has been observed in college students in other Asian countries, such as Japan [45], Korea [46], India [47], Pakistan[48], and Bangladesh [49]. Because these countries shared similar collectivist cultures such as an emphasis on family relationships, the family functioning may have an even greater severe impact on mental health during the pandemic compared with individualistic cultures.

It is worth noticing that after returning to school, the incidence rates of depression decreased while anxiety increased, a previous study has illustrated possible reasons for the deterioration in the mental health of students during home isolation, such as physical inactivity, lack of academic schedule, and increased reliance on digital use [50]. It is possible that after returning to school, students would have more space to do physical exercise, study more regularly, and return to a more organized life, all of which would helpful in reducing depressive symptoms, however, anxiety will rise due to back to a pre-pandemic academic schedule, which means more strict assignments and exams than the online learning period.

The result of the interaction effect of family functioning and time with MHP shows different family functioning group changes in the varying pattern through time: the incidence rates of both anxiety and depression rose during the stay-at-home period in all family groups, which indicates that even with HF family functioning may not fully protect the mental health of students from the impact of the pandemic. After students returned to school at T4, the direct influence of family functioning lessened and interaction with friends and classmates became normal, which may partly explain the reduction in the incidence rates of depression in HF and MdF groups after school reopened. However, the incidence rates of anxiety in the HF and MdF group increased after school reopened, according to X Wang, S Hegde, C Son, B Keller, A Smith and F Sasangohar [51], after most universities and colleges shifted to online learning mode from March to June 2020, the academic-related concerns due to the pandemic situation increased which was associated with anxiety. There was no significant change in the incidence rates of anxiety and depression in the SdF group after school reopened, indicating a possible prolonged negative influence of family dysfunction on mental health.

After considering coping styles, the variance of each subgroup began to emerge. Interestingly, for the negative coping style with HF subgroup, unlike with other subgroups, the incidence rates of anxiety tended to reduce during the stay-at-home period and rise after school reopened, showing a stronger protective effect of family. However, for the active coping style with SdF subgroup, the incidence rates of both anxiety and depression tended to rise during the stay-at-home period and reduce after school reopened, showing a stronger protective effect of the coping style. Additionally, the negative coping style with the dysfunctional family subgroup had the highest incidence rates of all MHP during all time, indicating that the risk of having MHP was 10 times higher than the active coping style with HF subgroup. Mutual influence of coping style and family functioning may behind this finding. Having a functional family may encourage a more beneficial coping style in students, for example, asking for advances from other family members, and these behaviors may also have a positive influence over family functioning. On the other hand, people with an active coping style may also cope actively with family issues which may lead to better family functioning. Such a positive feedback mechanism of coping style and family functioning can lead to relatively large differences in MHP. These results corroborate the findings of previous studies in this field [28, 32].

There are very few studies that consider the relationship between MHP and strong response or weak response coping style. Most of the previous studies using SCSQ have divided participants into only two categories and used the differential value between the standard score of active coping minus the standard score of negative coping to determine the tendency of individual coping styles. If the differential value was greater than 0, it indicated that the individual generally adopted an active coping style and vice versa [28]. This grouping is arbitrary because the two scores may be very close and an individual may use many coping strategies to overcome difficulties, and therefore we used the method mentioned in Sect. 2.3.3 to differentiate among coping styles. This gave us a chance to investigate the changing pattern of those who reacted strongly to pull through the pandemic and those who reacted weakly. The result is interesting: both groups have lower incidence rates of both anxiety and depression than the negative coping group, as explained by N Ntoumanis, J Edmunds and JL Duda [30] and RS Lazarus and S Folkman [31], a strong response coping style is aroused by threat appraisals of the stressor, while a weak response coping style is aroused by benign appraisals of the stressor. However, the change in the pattern of these two groups in different family functioning for depression and anxiety are alike and their AOR did not change as drastically as that of negative coping style, which could be aroused from harm–loss appraisals. Effective coping requires a fit between social context, situational appraisals, and choice of coping responses[30]. When variations in actual coping behavior do not result in a “fit” between situational factors and actual coping efforts, one may increase their emotional arousal to a level exceeding that which they can tolerate [51], the current pandemic has not had serious losses for most student, so harm–loss appraisal is an inappropriate response, when the time pass by, the negative effects of inappropriate appraisal will get stronger, this can explain why MHP shows a more stable pattern in the active, strong/weak response coping style group compared with negative coping style. Furthermore, we found that the incidence rates of depression among students with strong response coping style had decreased to the same state as at the beginning of the pandemic after they returned to school, and the trend was independent of family functioning, however, the incidence rates of anxiety among them would still higher than the beginning of the pandemic after the school reopened. There may be a mediator here, behavioral activation. A strong response coping style would activate one's various coping behaviors when facing adversity, and behavioral activation happens to be helpful for the improvement of depression, but it may not be effective for anxiety [52].

These findings suggest that college management staff need to adapt in several ways in the future. First, when the epidemic eases or home isolation over a period of time, let students choose whether come back to school or keep staying at home, especially students with SdF, if coming back to school is not possible, low-intensity intervention such as web-based consultation service or telephone hotline is recommended. Second, enhancing active coping styles such as the tendency to look for different ways to solve the problem and seeking advice from reliable people. Third, to reduce students’ harm—loss appraisal of the epidemic situation, correct knowledge of the situation needs to be promoted timely.

### Strengths and limitations

This study has several advantages: we prospectively followed up a large sample of college students in multiple waves from the first month of the COVID-19 pandemic until the reopening of schools. COVID-19—related MHP depressive symptoms and anxiety were repeatedly assessed with well-established scales. Moreover, the coping style of participants was divided into four categories to get a more detailed result. However, our study is not without limitations. First, our sample was collected via convenience sampling methods and may not be fully representative of the general population of college students in China. For example, females constituted a relatively large proportion of the sample, which might limit the generalizability of our results the reason for this may be that females are more responsive and cooperative in the context of the questionnaire survey [53, 54]. Second, current mental health disorders were collected by single self-reported items, but no structured or standardized clinical diagnostic interview was used to validate the diagnoses according to DSM or ICD criteria which may be affected by recall bias. Third, the coping style was measured at T1 and family functioning was measured at T2 because the development of the epidemic was not clear in the initial few months. Although SCSQ has high test–retest reliability (0.89) [37], a lack of repeated measurements could lead to bias in estimates. Also, due to different threat intensities and lifestyle challenges and possibilities, there are potential changes in coping styles throughout the study period that were not assessed. Lastly, the study is limited by the lack of information on online learning relating factors, such as satisfaction with online teaching, self-learning ability, etc., that may influence mental health status.

## Conclusion

The COVID-19 pandemic has had prolonged negative effects on the mental health of college students in China. Even students with HF or active coping style had a deterioration in their mental health. The negative influence of dysfunctional family functioning does not cease affecting the mental health of students even after schools restarted. MHP shows a more stable pattern for the active, strong/weak response coping style group compared with the negative coping style. These findings contribute to a greater understanding of the interaction of family functioning and coping style with the mental health of college students during the COVID-19 pandemic. It is now a well-established fact that pandemic-induced mental health issues have become a big global issue. It is therefore the need of the hour to pay more attention to students with dysfunctional family functioning and to help them cultivate active coping styles. Furthermore, based on the different needs of students depending on their family situation, to support students’ mental health it may be beneficial to let students choose whether come back to school or keep staying at home. Lastly, in the face of increased mental health challenges for students during the pandemic, it is recommended to provide them with alternative methods of support that remain accessible even without direct contact, such as web-based consultation service or telephone hotline.

## Supplementary Information

**Additional file 1 Table S1.** Pairwise comparisons of MHP incidence rates at different time point (%). **Table S2.** Pairwise comparisons of MHP incidence rates of different FF at different time point (%).**Table S3**. Pairwise comparisons of MHP incidence rates of different FF with different coping style at different time point (%). Supplemental file of Impact of family functioning on mental health problems of college students in China during COVID-19 pandemic and moderating role of coping style: a longitudinal study.

## Data Availability

The datasets used and/or analysed during the current study available from the corresponding author on reasonable request.
