# Supplementary material for: Impact of family functioning on mental health problems of college students in China during COVID-19 pandemic and moderating role of coping style: a longitudinal study
Source: BMC Psychiatry. 2023 Apr 12;23:244. doi: 10.1186/s12888-023-04717-9 (PMC10089825; doi:10.1186/s12888-023-04717-9)
Supplement: Supplementary file 1 — Additional file 1 Table S1. Pairwise comparisons of MHP incidence rates at different time point (%). Table S2. Pairwise comparisons of MHP incidence rates of different FF at different time point (%).Table S3. Pairwise comparisons of MHP incidence rates of different FF with different coping style at different time point (%). Supplemental file of Impact of family functioning on mental health problems of college students in China during COVID-19 pandemic and moderating role of coping style: a longitudinal study. [file 12888_2023_4717_MOESM1_ESM.docx]

Supplemental Tables shows pairwise comparisons of significant interaction effect of different MHP, T4-T3 represent the difference before and after resuming school, T3-T1 represent the variance of the home quarantine, T2-T1 represent the change of the first 2 mouth of home quarantine, T3-T2 represent the change of the last 2 mouth of home quarantine, T4-T1 represent the difference of starting home quarantine to resuming school. Unsignificant data was show by blank grid, girds with numbers are all significant at *p* < 0.05 level.

Table S1 Pairwise comparisons of MHP incidence rates at different time point (%)

| **MHP** | **T4-T3** | **T3-T1** | **T2-T1** | **T3-T2** | **T4-T1** | **T4-T2** |
| --- | --- | --- | --- | --- | --- | --- |
| Depression | -3.45 | 9.66 | 5.34 | 4.32 | 6.21 | - |
| Anxiety | 4.03 | 5.05 | 2.64 | 2.41 | 9.08 | 6.43 |

note: all numbers are all significant at *p* < 0.05 level.

Table S2 Pairwise comparisons of MHP incidence rates of different FF at different time point (%)

| **MHP** | **Family Function** | **T4-T3** | **T3-T1** | **T2-T1** | **T3-T2** | **T4-T1** | **T4-T2** |
| --- | --- | --- | --- | --- | --- | --- | --- |
| Depression | HF | -3.58 | 6.02 | - | 5.00 | 2.44 | - |
|  | MdF | -2.71 | 14.11 | 6.70 | 7.41 | 11.39 | 4.70 |
|  | SdF | - | 7.64 | 8.65 | - | 4.61 | -4.05 |
| Anxiety | HF | 3.45 | - | - | - | 4.62 | 4.42 |
|  | MdF | 3.93 | 11.70 | 5.50 | 6.20 | 15.62 | 10.13 |
|  | SdF | - | 4.03 | 3.61 | - | 7.61 | 4.00 |

note: all numbers are all significant at *p* < 0.05 level.

Table S3 Pairwise comparisons of MHP incidence rates of different FF with different coping style at different time point (%)

| **MHP** | **Family Function** | **Coping Style** | **T4-T3** | **T3-T1** | **T2-T1** | **T3-T2** | **T4-T1** | **T4-T2** |
| --- | --- | --- | --- | --- | --- | --- | --- | --- |
| Depression | HF | active | -2.82 | 8.37 | 2.38 | 6.00 | 5.55 | 3.17 |
|  |  | negative | -7.89 | - | - | 5.97 | - | - |
|  |  | strong response | -3.67 | 4.06 | - | 3.74 | - | - |
|  |  | weak response | - | 6.80 | - | 5.19 | 3.83 | - |
|  | MdF | active | - | 17.49 | 9.42 | 8.07 | 16.24 | 6.81 |
|  |  | negative | -4.34 | 10.18 | 3.81 | 6.37 | 5.85 | - |
|  |  | strong response | -3.90 | 15.74 | 6.73 | 9.01 | 11.85 | 5.12 |
|  |  | weak response | - | 14.40 | 7.25 | 7.15 | 12.86 | 5.61 |
|  | SdF | active | - | 29.76 | 25.12 | - | 23.62 | - |
|  |  | negative | - | - | - | - | - | - |
|  |  | strong response | - | 9.86 | 13.36 | - | - | -10.08 |
|  |  | weak response | - | - | - | - | - | - |
| Anxiety | HF | active | 2.27 | 1.58 | - | - | 3.84 | 3.29 |
|  |  | negative | - | - | - | - | - | - |
|  |  | strong response | 3.34 | - | - | - | 4.76 | 4.08 |
|  |  | weak response | 2.89 | 2.57 | - | - | 5.46 | 5.11 |
|  | MdF | active | 8.06 | 8.79 | 5.42 | 3.37 | 16.85 | 11.43 |
|  |  | negative | - | 10.76 | 4.23 | 6.53 | 13.89 | 9.66 |
|  |  | strong response | - | 16.65 | 7.32 | 9.33 | 17.76 | 10.45 |
|  |  | weak response | 4.26 | 10.13 | 4.82 | 5.31 | 14.38 | 9.57 |
|  | SdF | active | - | 13.43 | 10.15 | - | 14.25 | - |
|  |  | negative | - | - | - | - | - | - |
|  |  | strong response | - | - | - | - | 12.16 | - |
|  |  | weak response | - | - | - | - | - | - |

note: all numbers are all significant at *p* < 0.05 level.
